# Supplementary material for: Nonlinear optical heating of all-dielectric super-cavity: efficient light-to-heat conversion through giant thermorefractive bistability
Source: Nanophotonics. 2022 May 16;11(17):3981–91. doi: 10.1515/nanoph-2022-0074 (PMC11502051; doi:10.1515/nanoph-2022-0074)
Supplement: Supplementary file 1 — Supplementary Material Details [file j_nanoph-2022-0074_suppl_001.pdf]

Research Article

Daniil Ryabov, Olesiya Pashina, George Zograf, Sergey Makarov, Mihail Petrov*

Supplementary Information for the paper 'Nonlinear optical heating of all-dielectric super-cavity: efficient light-to-heat conversion through giant thermorefractive bistability'

https://doi.org/10.1515/sample-YYYY-XXXX
Received Month DD, YYYY; revised Month DD, YYYY; accepted Month DD, YYYY

S1 Hysteresis characteristics

In this section we perform the analysis of the hysteresis parameters and study their connection with the governing parameters of the system - pump intensity y_0, frequency detuning $\Delta\tilde{\omega}$ and thermooptical parameter b. Switching between the solution happens at the turning points A and B in Fig. 2 (a). The x-axis distance between them defines the sensitivity of switching upon the variation of the pump intensity, while while the y-axis distance shows the temperature the change between the two states. To the hysteresis parameter, let us rewrite Eq. 5 in the following form:

$$\begin{gathered} f = y \cdot \left[\Gamma^2 + (y - y_s)^2\right], \\ f(y) = \frac{y_0}{1+b^2}, \end{gathered} \tag{S1}$$

which is a third power equation and, therefore could have one, two or three solutions. The turning points can be found from the condition $f' = 0$ which gives the following values of the amplitudes:

$$\begin{gathered} y_A = \frac{1}{3}\left(2y_s + \sqrt{D}\right), \\ y_B = \frac{1}{3}\left(2y_s - \sqrt{D}\right), \\ D = y_s^2 - 3\Gamma^2, \end{gathered} \tag{S2}$$

and corresponds to A and B points in Fig. 2 (a) in the main text. It is quite clear from these formulas that the turning points existence is determined by the condition $D > 0$ whereas $D = 0$ defines the critical point O position from which two branches of the solution appear. It is also worth noticing that $D > 0$ condition imposes restrictions on the possible values of relative nonlinear coefficient $b = \beta/\alpha$ since for $b > b_{\rm cr} = 1/\sqrt{3}$ this requirement could be satisfied for only negative values of y_0 which in its turn should result into nonphysical negative incident intensities. Therefore, for large enough absorptive nonlinear coefficient $\beta > \alpha/\sqrt{3}$ bistability can not be observed.

Using Eq. S2 one can immediately find the 'height' of the hysteresis area which we define as the difference between amplitude values of the turning points:

Daniil Ryabov, Olesiya Pashina, George Zograf, Sergey Makarov, Mihail Petrov*, ITMO University, Department of Physics, Saint Petersburg, Russia

$$H_{\mathrm{h}} = y_A - y_B = \frac{2\sqrt{D}}{3}. \tag{S3}$$

Corresponding pump intensities y_0 should be then obtained by substituting mode amplitude values of turning points in Eq. S1:

$$\begin{aligned} y_0(A) &= f\left(y_A\right) \cdot \left(1+b^2\right) = \frac{2}{27}\left(y_s \cdot \left[y_s^2 + 9\Gamma^2\right] + D^{3/2}\right) \cdot \left(1+b^2\right) \\ y_0(B) &= f\left(y_B\right) \cdot \left(1+b^2\right) = \frac{2}{27}\left(y_s \cdot \left[y_s^2 + 9\Gamma^2\right] - D^{3/2}\right) \cdot \left(1+b^2\right), \end{aligned} \tag{S4}$$

which in its turn allows us to estimate hysteresis 'width' which we define as difference between pump intensities of turning points A and B.

$$W_{\mathrm{h}} = y_0(B) - y_0(A) = \frac{4D^{3/2}}{27} \cdot \left(1+b^2\right) = H_{\mathrm{h}}^3 \frac{1+b^2}{2} \tag{S5}$$

Interestingly, that both W_{h} and H_{h} tend to zero when $D \to 0$ which again proves merging of two solutions in critical point O and, hence, disappearance of bistability in the system.

For $b = 0$ these expressions could be written explicitly in a more simple way as a function of frequency detuning:

$$\begin{aligned} H_{\mathrm{h}} &= \frac{2}{3}\sqrt{\Delta\tilde{\omega}^2 - 3}; \\ W_{\mathrm{h}} &= \frac{H_{\mathrm{h}}^3}{2}, \end{aligned} \tag{S6}$$

from which it is clear that for large values of detuning $\Delta\tilde{\omega} \gg 1$ hysteresis area height has a linear dependence $H_{\mathrm{h}} \sim \Delta\tilde{\omega}$ and its width therefore cubic behaviour $W_{\mathrm{h}} \sim \Delta\tilde{\omega}^3$, whereas for close to the critical point values $\Delta\tilde{\omega} = \Delta\tilde{\omega}^* + \delta$ we have $H_{\mathrm{h}} \sim \delta^{1/2}$ and $W_{\mathrm{h}} \sim \delta^{3/2}$. Consequently, for small values of detuning near the critical point hysteresis height increases more rapidly than its width and the situation is opposite for large detuning values (see Fig. S1(a)),which should be considered while optimizing bistability area parameters. For nonzero thermooptical parameter b values behaviour of the system slightly changes which results mainly in critical point O shift towards higher frequency detunings (see Fig. S1(b,c)).

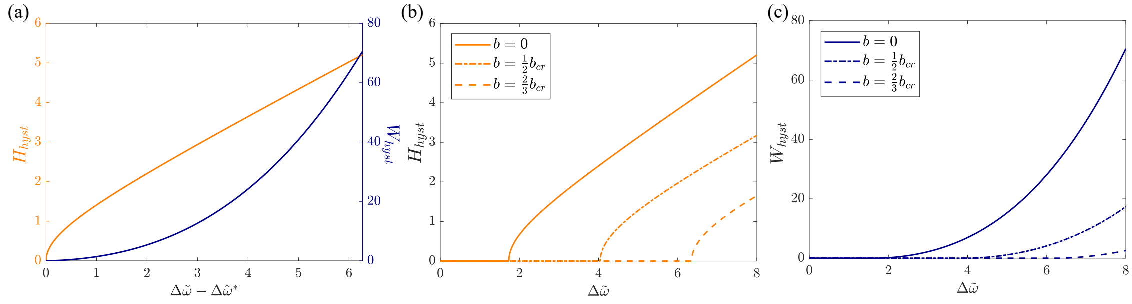

Fig. S1: (a) The dependence of hysteresis characteristics on detuning parameter $\Delta\tilde{\omega}$ for $b = 0$. (b,c) The dependence of hysteresis height H_{h} and width W_{h} on detuning parameter $\Delta\tilde{\omega}$ for different thermo-optical coefficients $b = 0, b = b_{cr}/2$ and $b = 2b_{cr}/3$ accordingly.
